# Exercises on Chapter Second

**Published:** 1883-08

**Authors:** 


### EXERCISES ON CHAPTER SECOND.

---

How was chemistry simplified by Berzelius?

What is a symbol in chemistry?

What language gives names to the chemical elements?

When several names of the elements begin with the same letter, how are they then distinguished?

Give examples?

How is quantivalence designated?

How many of the elements are interesting to the student of medicine and pharmacy?

Give the monad group of elements?

What is a monad?

Give the dyad group?

Which is the most important element in this group?

Give the triad group?

Which is the leading atom of this group?

Give the tetrad group?

Which is its leading element?

Give the hexad group?

Which is the most interesting element of this group?

Are there distinct pentad and heptad groups in pharmaceutical chemistry?

But are there not pentads and heptads in its category of elements?

Repeat the symbols of the monads?

The symbols of the dyads?

The symbols of the triads?

The symbols of the tetrads?

The symbols of the hexad?

Give the atomic weights of the monads?

The atomic weights of the dyads?

The atomic weights of the triads?

The atomic weights of the tetrads?

The atomic weights of the hexads?

How many functions has a symbol?

What are they?

How are atoms multiplied?

How are molecules multiplied?

Give examples of each?

What is a binary compound or molecule?

How is it formed?

Give an example?

Are the binary compounds numerous and important in chemistry?

What terminal always designates a binary?

By what rule are the constituents placed in the formula?

In writing the formula of water, why is the hydrogen atom doubled?

Give the fundamental rule of all chemical nomenclature?

Give the first rule for the formation of binaries?

What is the rule of construction for names of binaries?

Give examples?

What is the rule of modification in naming binaries?

Explain its relation to quantivalence?

Explain the use of the prefixes *hypo* and *per*?

How are molecules multiplied?

Give the rule for writing binary formulas when the quantivalence of the constituent is the same?

Give rule for the same when the quantivalences of the constituents are different?

Give examples?

Do the above rules apply to the formation of molecules of acids, bases and salts?

